# Squamous cell cancers contain a side population of stem-like cells that are made chemosensitive by ABC transporter blockade

**DOI:** 10.1038/sj.bjc.6604185

**Published:** 2008-01-22

**Authors:** M R Loebinger, A Giangreco, K R Groot, L Prichard, K Allen, C Simpson, L Bazley, N Navani, S Tibrewal, D Davies, S M Janes

**Affiliations:** 1 Centre For Respiratory Research, Rayne Institute, University College London, 5 University Street, London WC1E 6JJ, UK; 2 Keratinocyte Laboratory, Cancer Research UK, Cambridge Research Institute, Robinson Way, Cambridge CB2 0RE, UK; 3 Flow Cytometry Laboratory, Cancer Research UK, London Research Institute, 44 Lincoln's Inn Fields, London WC2A 3PX, UK

**Keywords:** stem cell, squamous cell carcinoma, side population, ATP-binding cassette transporters

## Abstract

Cancers are a heterogeneous mix of cells, some of which exhibit cancer stem cell-like characteristics including ATP-dependent drug efflux and elevated tumorigenic potential. To determine whether aerodigestive squamous cell carcinomas (SCCs) contain a subpopulation of cancer stem cell-like cells, we performed Hoechst dye efflux assays using four independent cell lines. Results revealed the presence of a rare, drug effluxing stem cell-like side population (SP) of cells within all cell lines tested (SCC-SP cells). These cells resembled previously characterised epithelial stem cells, and SCC-SP cell abundance was positively correlated with overall cellular density and individual cell quiescence. Serial SCC-SP fractionation and passaging increased their relative abundance within the total cell population. Purified SCC-SP cells also exhibited increased clonogenic potential in secondary cultures and enhanced tumorigenicity *in vivo*. Despite this, SCC-SP cells remained chemotherapeutically sensitive upon ATP-dependent transporter inhibition. Overall, these findings suggest that the existence of ATP transporter-dependent cancer stem-like cells may be relatively common, particularly within established tumours. Future chemotherapeutic strategies should therefore consider coupling identification and targeting of this potential stem cell-like population with standard treatment methodologies.

Stem cells are present in many tissues where they function to maintain appropriate homeostasis and differentiation as well as regulate tissue responses to injury and infection (Watt and Hogan, 2000; Janes *et al*, 2002). These cells are capable of self-renewal, have a relatively undifferentiated phenotype, exhibit high proliferative potential, and are multipotent (Griffiths *et al*, 2005). In adult tissues, stem cells tend to divide infrequently and many exhibit an intrinsic resistance to environmental toxins or chemotherapeutic agents (Giangreco *et al*, 2002; Zhou *et al*, 2002; Hirschmann-Jax *et al*, 2004).

Traditionally, stem cell pollutant resistance has been identified on the basis of efficient Hoechst 33342 dye efflux (Goodell *et al*, 1996). Drug-resistant stem cells, termed side population (SP) cells, have been identified in a growing number of tissues including bone marrow, pancreas, muscle, brain, and lung (Goodell *et al*, 1996; Lechner *et al*, 2002; Giangreco *et al*, 2003; Summer *et al*, 2003; Kondo *et al*, 2004; Oyama *et al*, 2007). This SP phenotype is dependent on ATP-binding cassette (ABC) transporter activity, as ATP inhibitors such as verapamil and reserpine block dye efflux (Goodell *et al*, 1997; Zhou *et al*, 2001; Scharenberg *et al*, 2002). In particular, the ABC transporter ABCG2/BCRP1 appears critical to maintain this phenotype as Abcg2 knockout mice lack SP cells and are particularly sensitive to chemotherapeutic agents such as mitoxantrone (Zhou *et al*, 2002).

In addition to normal tissues, recent evidence suggests that some cancers also contain a population of stem-like, pollutant-resistant cells (Passegue *et al*, 2003). Cancer stem or stem-like cells, which exhibit increased chemotherapeutic drug resistance, have been found within acute myeloid leukaemia (Wulf *et al*, 2001; Reya *et al*, 2003), breast, and brain solid tumours (Al-Hajj *et al*, 2003). Recently, several cancer cell lines have been identified that contain a subpopulation of chemotherapeutic-resistant, cancer SP cells including the rat glioma line C6, human breast cancer line MCF-7, rat neuroblastoma line B104, and the human adenocarcinoma cell line HeLa (Kondo *et al*, 2004). Cancer SP cells have also been identified *in vivo* within 65% of human neuroblastoma tumours (Hirschmann-Jax *et al*, 2004). These repopulate both SP and non-SP cell types, indicating their multipotent differentiation potential (Hirschmann-Jax *et al*, 2004). They also express numerous ABC transporter proteins, providing them with increased drug resistance (Hirschmann-Jax *et al*, 2004). Finally, it appears that following chemotherapy, cancer SP are uniquely capable of producing drug-insensitive secondary tumours (Passegue *et al*, 2003). On the basis of these observations, cancer SP cells are increasingly considered to be potential cancer stem cells (Hadnagy *et al*, 2006; Giangreco *et al*, 2006).

Aerodigestive squamous cell carcinomas (SCCs) frequently exhibit chemotherapeutic resistance and increased metastatic potential following unsuccessful cancer treatment. We, therefore, wished to determine whether SCC cell lines contained SP cells, and, if so, whether these displayed cancer stem cell characteristics. We now describe the presence of an SCC-SP cell subset whose abundance was positively correlated with increasing cell confluence and quiescence. These cells exhibited characteristics of cancer stem cells including increased growth, multipotent differentiation, and a capacity for *in vivo* tumorigenesis.

## MATERIALS AND METHODS

### Tissue culture

H357, SCC4, SCC13, and SCC15 cell lines, derived from patients with advanced SCCs, were cultured in complete FAD medium (Sugiyama *et al*, 1993). The culture medium (FAD+FCS+HICE) consisted of one part Ham's F12 medium and three parts Dulbecco's modified Eagle's medium, supplemented with 10% fetal calf serum (FCS), 0.5 *μ*g ml^−1^ hydrocortisone, 5 *μ*g ml^−1^ insulin, 10^−10^ M cholera toxin, and 10 ng ml^−1^ epidermal growth factor (HICE). The culture medium was changed every 2 days.

### Fluorescence-activated cell sorting

Cells were harvested at 70% or full confluence, washed, and resuspended at 1 × 10^6^ cells ml^−1^ in FAD medium and incubated at 37°C for 10 min. Cells were labelled with 5 *μ*M Hoechst 33342 (Sigma, St Louis, MI) for 15, 30, 45, 60, 75, 90, 105, 120, and 135 min at 37°C to determine the required incubation time. As the size of the SP was found to be stable from 30 min, all subsequent staining was carried out for 45 min at 37°C. The cells were counterstained with 5 *μ*g ml^−1^ propidium iodide (Sigma) to label dead cells, which were excluded from the analysis. Fluorescence-activated cell sorting (FACS) was performed using a MoFlo High-Performance Cell Sorter (DakoCytomation, Denmark). For SP analysis, at least 1 × 10^5^ total events were collected, and all subsequent analysis was performed using FlowJo software (Tree Star Inc., Ashland, Oregon). For cell cycle status analysis following Hoechst 33342 efflux, ethanol-fixed cells were stained with 50 *μ*g ml^−1^ propidium iodide in the presence of RNase A (50 *μ*g ml^−1^) and analysed with a FACS-Calibur flow cytometer. The Watson Pragmatic model was applied to determine cell cycle. All flow cytometry experiments were repeated to confirm consistency. A representative plot is shown for each profile.

### Colony-forming assays

In all, 200 SP and non-SP H357 cells were seeded per six-well plate and cultured for 14 days. Colonies were washed, fixed using 3% paraformaldehyde (BDH), and stained with Rhodanile Blue overnight. Colonies of two or more cells were counted using an Olympus CK2 inverted phase-contrast light microscope. Abortive colonies were defined as colonies that contained fewer than 32 cells according to the system described by Jones and Watt (1993). A large colony was defined as greater than 32 cells per colony. All experiments were performed in triplicate. In mitoxantrone dihydrochloride (Sigma) dose–response assays, 200 H357 cells were plated per well of a six-well plate and cultured in the presence of 0, 1, or 10 ng ml^−1^ of mitoxantrone for 3 days. After a further 14 days, the cultures were fixed, stained, and counted. In mitoxantrone and verapamil assays, 300 H357 cells were plated per well of a six-well plate, cultured in the presence of 0, 1, or 10 ng ml^−1^ of mitoxantrone±verapamil hydrochloride 100 *μ*M (Sigma) for 7 days, and, subsequently, grown for a further 7 days in the absence of drugs.

### Proliferation assay

A total of 1000 H357 cells were plated per well and cultured in complete FAD medium. Cells were harvested at days 2, 4, and 8, and cell number counted by haemocytometer. Each H357 cell population and time point were analysed in triplicate.

### Quantitative RT-PCR

cDNA synthesis (random hexamers+Superscript II; Invitrogen, Paisley, UK) and subsequent quantitative RT-PCR (QPCR) was performed using 1 *μ*g of total RNA isolated from freshly sorted or serially passaged H357 SP, non-SP, and parent cells as indicated in text. Human gene-specific, predesigned, and inventoried probes were purchased from Applied Biosystems, Foster City, CA, TaqMan QPCR analysis was based on the ΔΔ*C*_t_ relative mRNA abundance method and normalised to *β*2-microglobulin expression. At least two samples per sort parameter were assayed using an ABI7900 real-time PCR machine (Applied Biosystems).

### Subcutaneous tumour model

Six-week-old, male NOD/SCID mice purchased from Harlan (Bicester, UK) were used for the experiments. All mouse studies were performed in accordance with British Home Office procedural and ethical guidelines. Animals were housed in pathogen-free conditions with filtered air, and autoclaved food and water was available *ad libitum*. H357 SP(1) and G2(1) cells (both sorted and passaged once to expand cell numbers) were suspended in sterile PBS at a concentration of 1 × 10^7^ cells ml^−1^. A total of 200 *μ*l of the suspension containing two million cells was injected subcutaneously in the left flank with a 29G needle. Tumours were measured every 3–5 days with callipers, and the volume calculated as 4/3 *πr*^3^, where *r* is the estimated radius.

### Statistics

Student's *t*-, Student–Newman–Keuls, and Mann–Whitney statistical tests were carried out using SigmaStat. Data are expressed as means±s.e.m. Data not in normal distribution were log transformed prior to statistical testing.

## RESULTS

### Squamous cell carcinomas contain an ABC transporter- and cell density-dependent side population

**Figure 1:**
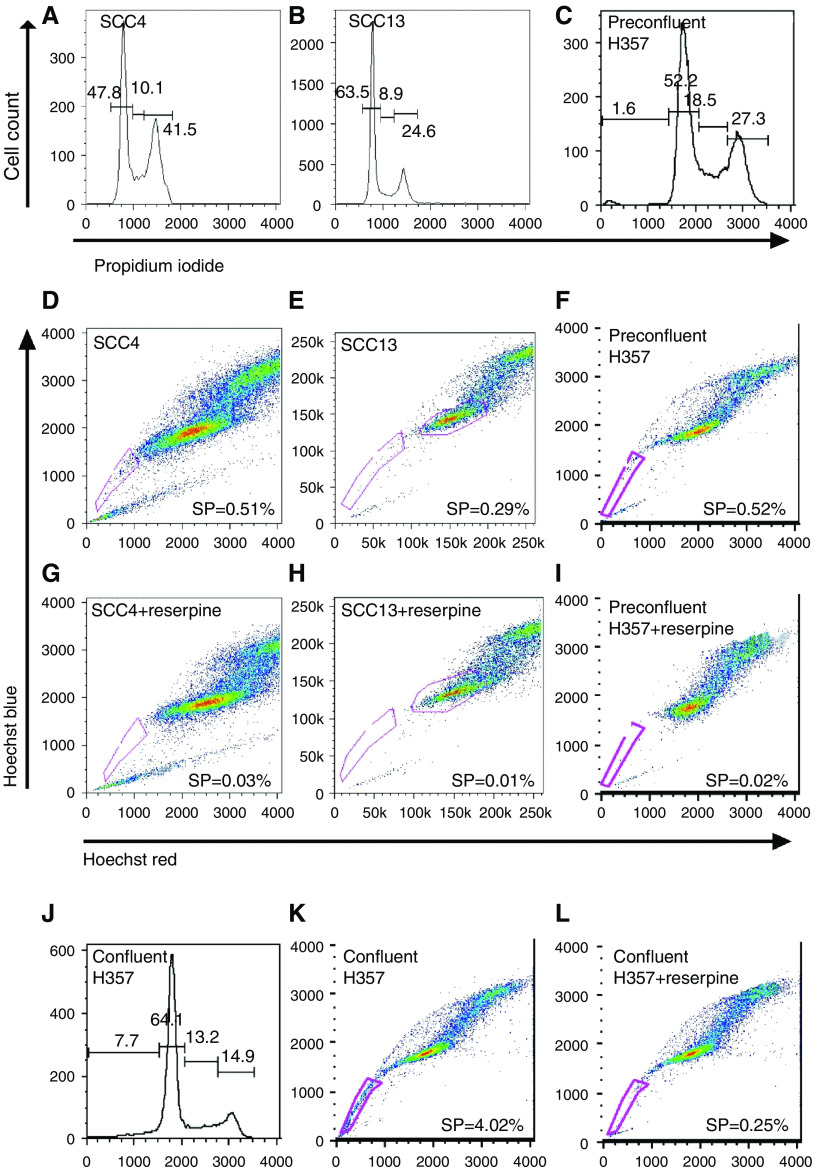
Squamous cell carcinomas contain an SP that varies with cell confluency. Seventy percent confluent SCC4, SCC13, and H357 SCC cells were stained with propidium iodide staining profiles, highlighting the relative percentage of S-phase cells as an indication of cell confluence (**A**–**C**, **J**). The cells were incubated in Hoechst 33342 prior to analysis by flow cytometry (**D**–**F**). The SP was compared between subconfluent (70%) (**F**) and confluent (**K**) cell populations with reserpine controls (**G**–**I**, **L**).

Several tumours and tumour cell lines maintain a population of chemotherapeutic and pollutant-resistant cells with characteristics of stem-like SP cells (Hirschmann-Jax *et al*, 2004; Kondo *et al*, 2004). To determine whether SCCs might also contain a subpopulation of drug-resistant SP cells, four 70% confluent SCC cell lines were incubated in 5 *μ*M Hoechst 33342 dye and analysed by FACS. Cell confluence was verified by cell cycle analysis (Figures 1A–C). A characteristic SP fraction was detected in all four cell lines examined and was stable after 30 min dye incubation as determined by time course analysis (15–135 min, SP examined every 15 min; not shown). Overall, SCC-SP abundance varied between each cell line examined from 0.076% (SCC15) to 0.47% (H357) (Figures 1D–F and Table 1). All SCC-SP populations were reserpine sensitive, indicating their dependence on ABC-type transporter activity (Figures 1G–I).

We next wished to examine whether a cancer's cellular density or cell cycle status influenced SCC-SP cell abundance. We therefore cultured H357 cells to 100% confluence prior to Hoechst dye incubation (Figure 1J). On average, increased cellular density or reduced proliferation increased the H357 SP cell population from 0.47±0.16 to 4.53±0.61%, or nearly 10-fold (*n*=4, Figures 1K and L).

### SCC-SP cells express epithelial stem cell markers

**Figure 2:**
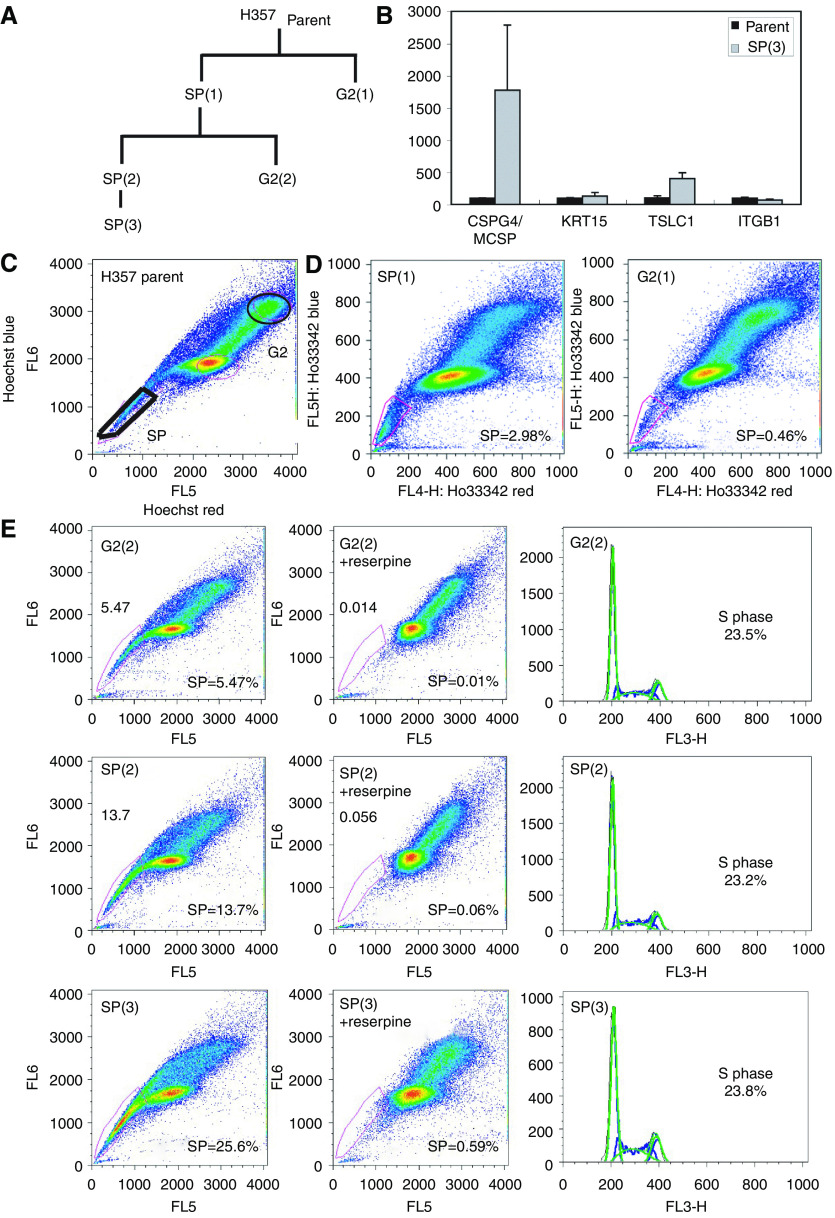
Squamous cell carcinomas containing SP cells express stem cell markers and are selectable. (**A**) Schematic of H357 cell populations isolated following successive rounds of FACS analysis. (**B**) Relative mRNA abundance of epithelial stem cell markers chondroitin sulphate proteoglycan 4 (CSPG4/MCSP), tumour suppressor of lung cancer 1 (TSLC1), integrin *β*1 (ITGB1), and keratin 15 (KRT15). (**C**) FACS analysis of Hoechst 33342-stained parental H357 cells with sorted areas marked SP and G2. (**D**) FACS analysis of Hoechst 33342-stained cell populations isolated from (**C**). (**E**) G2(2) and SP(2) cells were stained with Hoechst 33342 and analysed in the presence or absence of reserpine. Side population size is increased in SP(2) cells and further in SP(3) cells, suggesting that the SP phenotype is selectable. Propidium iodide staining indicates equivalent levels of confluence and cell viability.

To ascertain whether SCC-SP cells exhibited an enhanced stem cell-like phenotype, we isolated and serially propagated H357 SCC-SP cells as indicated in Figure 2A. Using QPCR, we examined the expression of known epithelial stem cell genes including chondroitin sulphate proteoglycan 4 (CSPG4/MCSP; Legg *et al*, 2003), tumour suppressor of lung cancer 1 (TSLC1; Morris *et al*, 2004; Tumbar *et al*, 2004), integrin *β*1 (ITGB1; Jones and Watt, 1993), and keratin 15 (KRT15; Liu *et al*, 2003). Serially propagated SCC-SP cells (SP(3)) expressed significantly more CSPG4/MCSP and TSLC1 than parent H357 cells but did not express increased levels of either KRT15 or ITGB1 (Figure 2B). In freshly isolated, unpassaged SP(1) cells, only TSLC1 expression was significantly enriched relative to parent cells (data not shown). Altogether, these data indicate that SCC-SP cells but not parent H357 cells resemble epithelial stem cells.

### Serial SCC-SP cell propagation enhances SP cell abundance

We next wished to examine whether isolated and *in vitro* expanded H357 SP cells were capable of repopulating secondary SP and non-SP populations. Parent H357 cells were therefore labelled with Hoechst 33342 dye and sorted into two populations termed SP(1) and G2(1) based on their relative blue/red fluorescence intensity (Figure 2C, schematic Figure 2A). The G2 grouping reflected each cell's relative position in the cell cycle (that is, G2 phase; Figure 2C). Following *in vitro* expansion, G2 cells reproduced both an SP and non-SP cell population in similar proportions to those of the parent population (0.46%, Figure 2D). In contrast, SP(1)-sorted cells produced greater numbers of drug-resistant SP cells relative to both parent and G2 populations (2.98%; Figure 2D).

On the basis of our observation that SCC-SP cell expansion leads to an enrichment of drug-resistant cells, we wished to determine whether further SP cell propagation might continue to increase SP cell abundance. H357 SP(1) cells were therefore re-sorted into SP(2) and G2(2) fractions; SP(2) was additionally expanded and fractionated into an SP(3) population (see schematic, Figure 2A). Under these conditions, the ABC-dependent drug-resistant SP fraction increased dramatically within both SP(2)- and G2(2)-sorted subpopulations compared to the original parent cells (Figure 2E). Further SP(2) fractionation to SP(3) increased the SP fraction to 25.6% of total cells, an increase of approximately 50-fold from the original parental population (Figure 2E). These percentages were confirmed with a repeat experiment (data not shown).

### SCC-SP cells express ABCG2 and ABCC1 type ABC transporters

**Figure 3:**
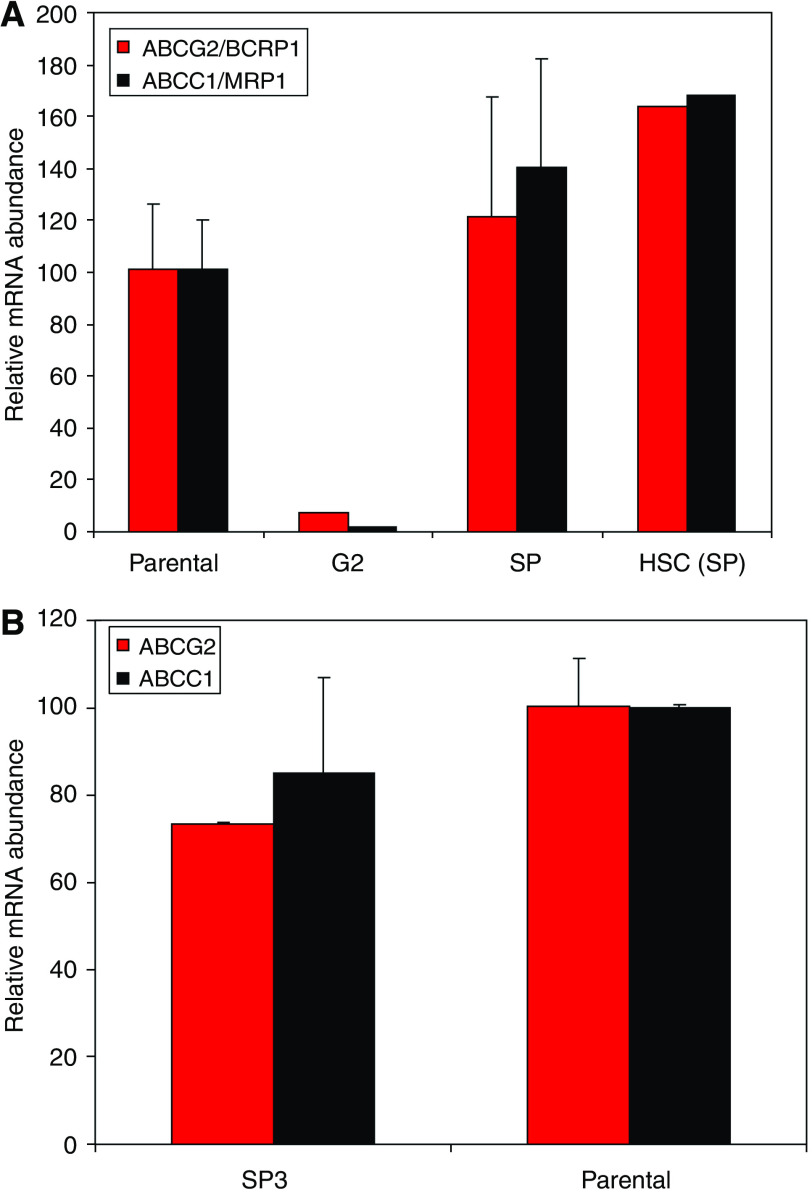
Quantitative RT-PCR of SCC populations. (**A**) Unpassaged SP(1) and G2(1) cells show differential expression of ABCG2 and ABCC1 transporter proteins. SP(1) levels are equivalent to parent and haematopoietic stem cells. (**B**) Highly selected SP(3) cells have equivalent expression to parent cells.

On the basis of the observation that SCC-SP purification enriched the relative SP abundance in secondary cultures, we wished to examine whether SP cells also contained elevated ABC transporter expression relative to parent and non-SP cells. Results of QPCR analysis revealed that both parent and freshly sorted SP(1) but not G2 cells expressed ABCG2(BCRP1) and ABCC1(MRP1) transporters (Figure 3A). Expression levels were not significantly different from those of independently isolated haematopoietic SP stem cells (HSC (SP), Figure 3A). There was additionally no significant difference in either ABCG2 and ABCC1 expression when serially propagated SP(3) cells were compared with parent SCCs (Figure 3B). We were unable to detect ABCB1/MDR1 expression in any SCC cell lines examined. Overall, these results suggested that ABC transporter mRNA expression does not directly determine SP cell abundance.

### SCC-SP cells exhibit stem cell-like characteristics *in vitro*

**Figure 4:**
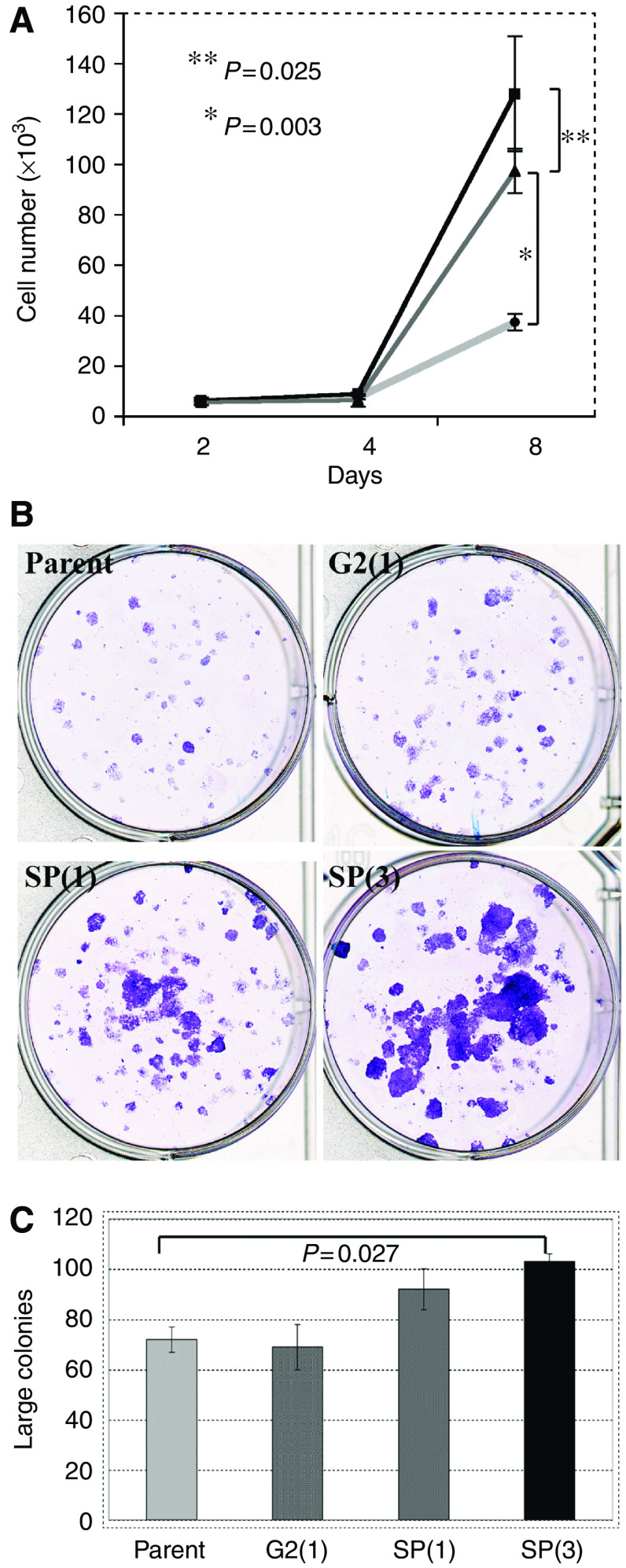
Side population cells have higher proliferation rates and greater clonogenic ability. (**A**) 1000 parent H357, SP(1), and SP(3) cells were plated and the total cell numbers counted at days 2, 4, and 8; SP(3) (squares), SP(1) (triangles), parent (circles). (**B**) Analysis of clonogenicity of H357 cell populations. (**C**) Quantitation of large colony numbers from clonogenicity assays shown in (**B**). Assays were set up in triplicate. Error bars indicate s.e.m.

One of the principal characteristics of epithelial stem cells is enhanced *in vitro* clonogenicity and growth. To examine whether SCC-SP cells exhibit enhanced *in vitro* proliferation, 1000 parent, SP(1), and SP(3) cells were cultured for 2, 4, and 8 days. Both SP cell populations grew significantly more rapidly than parent cells (Figure 4A). SP(3) cells also exhibited significantly faster growth rate 8 days postplating than SP(1) cells, indicating a serial enrichment in SCC-SP proliferative capacity (Figure 4A). The data shown are representative of two proliferation assays performed in triplicate.

We also examined the clonogenic potential of SCC-SP cells as an indicator of their individual proliferative capacity. Parent, G2(1), SP(1), and SP(3) H357 cells were plated in triplicate and cultured at clonal density for 14 days (Figure 4B). Results revealed significant increases in large colony formation uniquely within both SP(1) and SP(3) populations but not G2 cell populations when compared with parent cells (Figures 4B and C). These findings were especially evident following serial SP cell propagation (SP(3); Figures 4B and C).

### SCC-SP cells exhibit stem cell-like characteristics *in vivo*

**Figure 5:**
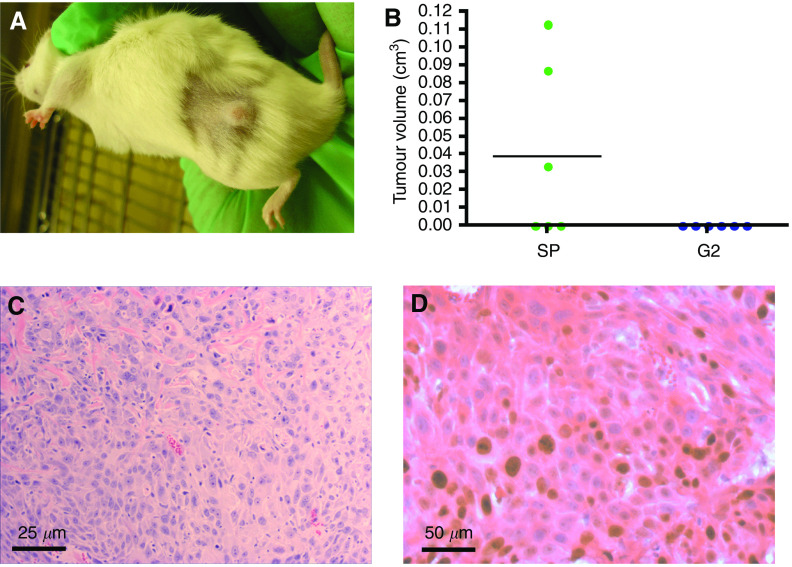
Squamous cell carcinomas containing SP cells are tumorigenic *in vivo*. (**A**, **B**) Three out of six NOD/SCID mice grew subcutaneous tumours following injection of two million SCC-SP(1) cells compared with no SCC G2(1) cell-derived tumours (*n*=6). (**C**) H&E confirmed SCC and (**D**) Ki67 staining demonstrated high cell proliferation.

To test for *in vivo* tumorigenic potential, we compared the tumour formation capacity of H357 SCC-SP and non-SP cells. We subcutaneously injected two million SP(1) cells (*n*=6) or two million G2(1) H357 cells (*n*=6) into the flank of NOD/SCID mice and allowed these to grow for 49 days. In accordance with previous attempts to grow H357 cells in an *in vivo* model (Jones *et al*, 1996; Janes and Watt, 2004), none of the G2(1) cell grafts produced tumours (Figures 5A and B). Interestingly, three out of six SCC-SP-sorted and -engrafted cell populations did produce subcutaneous tumours with an average volume of 0.038 cm^3^ (Figures 5A and B). Human SCC cell contribution to tumours and active cell proliferation were confirmed by H&E and Ki67 staining (Figures 5C and D). To our knowledge, this is the first demonstration of the ability of an H357 cell population to produce tumours *in vivo*, strongly suggesting that our SCC-SP contains a unique population of cancer stem-like cells.

### Stem-like SCC-SP cells are chemotherapeutic resistant but sensitised by verapamil

**Figure 6:**
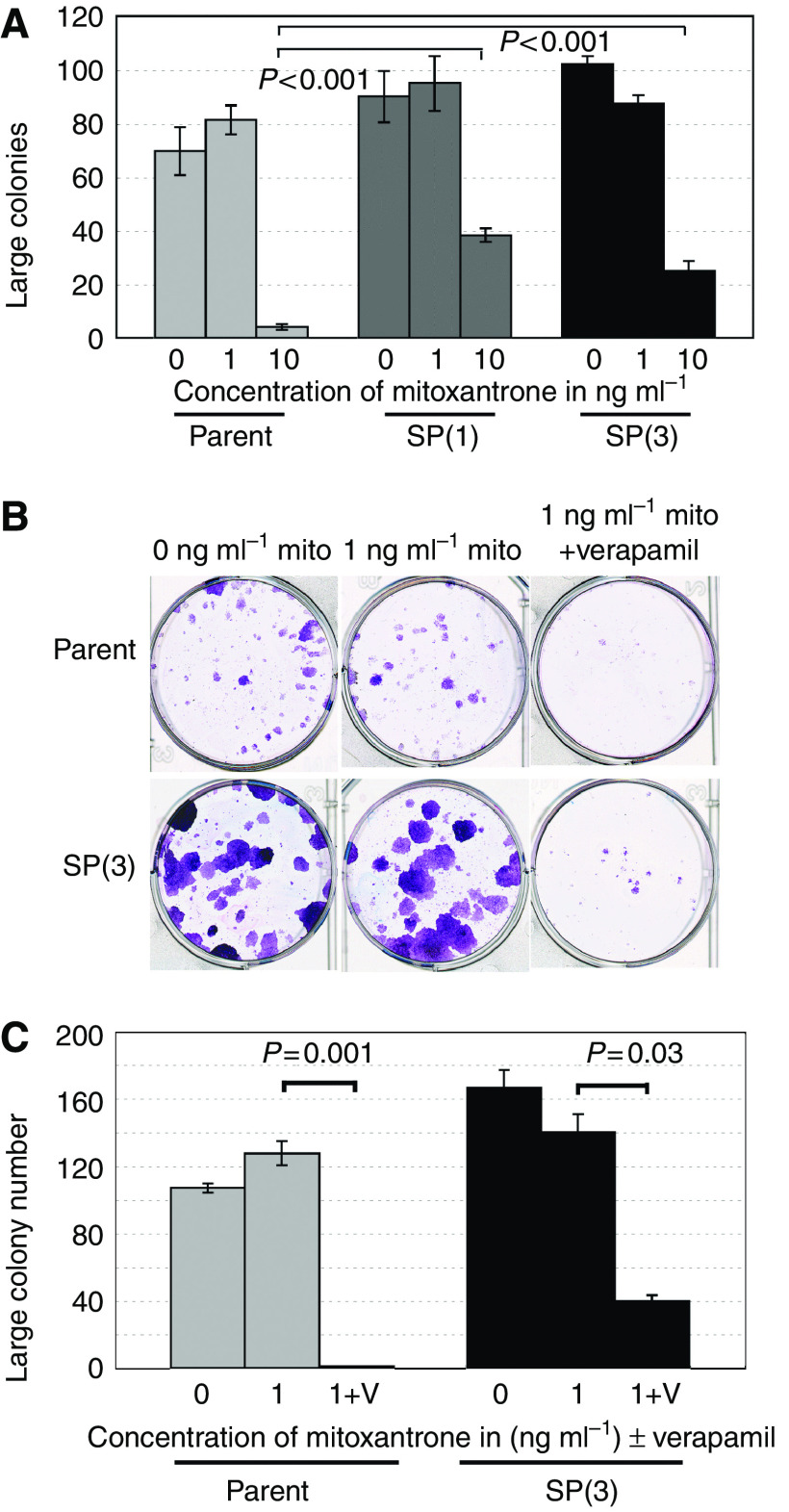
Side population cells have an increased level of resistance to mitoxantrone but are sensitised by concurrent ABC transporter blockade. (**A**) Analysis of stem cell colony numbers in clonogenicity assays of parent, SP(1), and SP(3) cells in the absence or presence of mitoxantrone at 1 or 10 ng ml^−1^. Cells were grown in the drug for 3 days and grown for a further 14 days in the absence of the drug before analysis. Assays were performed in triplicate. Error bars indicate s.e.m. (**B**) Parental and SP(3) cells were grown in 0 or 1 ng ml^−1^ mitoxantrone±verapamil for 7 days and, subsequently, grown for a further 7 days in the absence of drugs and clonogenicity analysed. (**C**) Quantitation of large colony numbers from (**B**). Assays were set up in triplicate. Error bars indicate s.e.m.

Our observation that SCC-SP cells expressed both ABCG2 and ABCC1 multidrug transporters and exhibited elevated Hoechst 33342 efflux indicated that these cells likely possessed resistance to cytotoxic chemotherapeutic drugs including mitoxantrone. To assess SCC-SP and parent cell mitoxantrone sensitivity, 200 cells were cultured in the presence of 0, 1, and 10 ng ml^−1^ mitoxantrone for 3 days followed by 14 days culture in mitoxantrone-free media. Results indicated that both SP(1) and SP(3) were significantly more resistant to 10 ng ml^−1^ mitoxantrone treatment and capable of maintaining large colony formation in comparison with parent cells (Figure 6A). All cells appeared resistant to 1 ng ml^−1^ mitoxantrone in agreement with previous ABC transporter expression data (Figure 3A).

To investigate whether the addition of ABC-dependent transport inhibitors might block mitoxantrone resistance and restore chemotherapeutic sensitivity, parent and SP(3) cells were cultured at clonal density in the presence of 1 ng ml^−1^ mitoxantrone alone or in combination with verapamil for 7 days followed by 7 days in the absence of both drugs. We found that both parent and SP(3) cells that previously exhibited full resistance to 1 ng ml^−1^ mitoxantrone were dramatically growth inhibited following verapamil treatment (Figures 6B and C). These data suggest that verapamil-dependent ABC transporter inhibition blocks efficient SCC-SP mitoxantrone efflux, thereby restoring chemotherapeutic drug sensitivity to this previously resistant and aggressive tumour stem cell-like population.

## DISCUSSION

The present study is the first to identify a Hoechst 33342 effluxing cell SP in several SCC cell lines that exhibits properties consistent with cancer stem or stem-like cells. The abundance of this SCC-SP varied between different SCC cell lines, and appeared highly dependent on both cellular density and proliferation status. As expected, SCC-SP cells expressed several previously characterised multidrug effluxing ABC-type transporters and were resistant to the chemotherapeutic agent mitoxantrone. SCC-SP cells also expressed elevated levels of the epithelial stem cell genes MCSP and TSLC1 relative to parent H357 cells. Importantly, we demonstrated that SCC-SP cells may be chemosensitised following the inclusion of ABC transport inhibitors. This finding may suggest novel approaches to cancer treatment, which could include the specific targeting of cancer stem cells.

Our observation that SCC-SP abundance is cellular density or confluence dependent has not been previously described. This observation may partially explain differences reported between several laboratories studying SP cells and highlights the importance of considering cell confluence when examining SP cells *in vitro*. It is also possible that these cellular density effects might contribute to the relative ‘stemness’ of cancer cells contained within an *in vivo* tumour microenvironment. For example, infrequently proliferating cells within well-established tumours might be able to divert increased resources towards maintaining this chemoresistant or stem cell-like phenotype.

Previous studies of cancers *in vitro* and primary tumours *in vivo* have shown that SP cells are uniquely capable of generating both SP and non-SP cell fractions. This data have been used to suggest that SP cells are multipotent ‘cancer stem cells’ (Hirschmann-Jax *et al*, 2004; Kondo *et al*, 2004). In the current study, we find that serial SCC-SP cell propagation selects for cells that generate an increased SP cell abundance while maintaining multipotent differentiation. This is in contrast to non-SP (G2) cell populations, which could only produce secondary cultures similar to the original parental H357 cell line. We therefore believe that our SCC-SP cells uniquely represent a cancer stem or stem-like cell population.

In addition to stem cell-like properties in SCC-SP cells including multipotent differentiation and high *in vitro*/*in vivo* growth capacity, we have also now shown that SCC-SP stem cell-like cells are rendered chemosensitive using simple ABC transport inhibitors including verapamil and reserpine. This finding is significant, as it has recently been observed that many cancers maintain subpopulations of stem-like cells, which are chemotherapy insensitive and uniquely maintain tumour regrowth capabilities (Bonnet and Dick, 1997; Al-Hajj *et al*, 2003; Passegue *et al*, 2003; Hirschmann-Jax *et al*, 2004; Singh *et al*, 2004; Patrawala *et al*, 2006). Thus, it is increasingly important that cancer treatments target and eradicate putative cancer stem cells to halt clinical tumour recurrence. Intriguingly, early combinatorial therapy experiments demonstrated an effective synergistic therapeutic effect using verapamil plus mitoxantrone administration in the treatment of ovarian cancer (Tsuruo *et al*, 1985; Hendrick *et al*, 1991). Whether or not this *in vivo* chemosensitisation occurred via similar mechanisms to those described in our current study is not known, although clearly these findings highlight the need for more research into this potentially combined therapy.

## Figures and Tables

**Table 1:** Summary of average SCC SP cell abundance

**Cell line**	**Average % of SP**	**s.d.**	**Minimum/maximum % of SP**	**No. of replicates**
H357	0.470	0.160	0.25/0.63	4
SCC4	0.385	0.177	NA	2
SCC13	0.270	0.028	NA	2
SCC15	0.076	0.063	NA	2

NA=not applicable; SCC=squamous cell carcinoma; SP=side population.

SP analysis performed at 70% cell confluence.
